# Time-Aware Explainable Recommendation via Updating Enabled Online Prediction

**DOI:** 10.3390/e24111639

**Published:** 2022-11-11

**Authors:** Tianming Jiang, Jiangfeng Zeng

**Affiliations:** School of Information Management, Central China Normal University, Wuhan 430079, China

**Keywords:** explainable recommendation, data leakage, model aging, online prediction, model updating

## Abstract

There has been growing attention on explainable recommendation that is able to provide high-quality results as well as intuitive explanations. However, most existing studies use offline prediction strategies where recommender systems are trained once while used forever, which ignores the dynamic and evolving nature of user–item interactions. There are two main issues with these methods. First, their random dataset split setting will result in data leakage that knowledge should not be known at the time of training is utilized. Second, the dynamic characteristics of user preferences are overlooked, resulting in a model aging issue where the model’s performance degrades along with time. In this paper, we propose an updating enabled online prediction framework for the time-aware explainable recommendation. Specifically, we propose an online prediction scheme to eliminate the data leakage issue and two novel updating strategies to relieve the model aging issue. Moreover, we conduct extensive experiments on four real-world datasets to evaluate the effectiveness of our proposed methods. Compared with the state-of-the-art, our time-aware approach achieves higher accuracy results and more convincing explanations for the entire lifetime of recommendation systems, i.e., both the initial period and the long-term usage.

## 1. Introduction

With the rapid development of the Internet, massive information results in an information overload problem, which makes it difficult for people to find the desired ones. On par with search engines, recommender systems are able to relieve this problem by modeling user preferences based on collected historical data [1]. According to the used data, recommendation models can be mainly categorized into collaborative filtering models [2,3], content-enriched models [4,5], and context-enriched models [6,7]. Collaborative filtering models render recommendations based on the similarity of users or items from the user–item interactions history. However, collaborative filtering models usually suffer from the data sparsity issue and the cold-start issue, which limit the recommendation performance [8,9]. Specifically, the data sparsity issue arises due to user interactions with a small portion of items, and the cold-start issue is due to the deficient information about new entities, i.e., new items or new users [10].

To this end, content-enriched models are proposed. For content-enriched models, besides user–item interactions history, content information, i.e., side information associated with users and items, is used as supplementary sources to catch more interaction details. Knowledge Graphs (KGs) or Heterogeneous Information Graphs (HIGs) are typical representation methods for content information organization, in which nodes are entities or attributes and edges are their relations [11]. Thanks to enriched content, the data sparsity issue and cold-start issue are alleviated and higher recommendation accuracy together with certain explainability are achieved [6,12].

However, most existing KG-based recommendation techniques use static knowledge graph with offline prediction in which models are trained once while used forever [13,14]. There are two main issues of these methods, namely the data leakage issue and the model aging issue. First, their dataset split settings shuffle dataset randomly without considering chronological order. Therefore, knowledge should not be known at the time of training is utilized, that is, data leakage occurs. As a result, data leakage leads to unrealistic high accuracy and unreasonable explainability, which cannot translate directly into good performance in a real world production scenario [15].

Second, the dynamic characteristics of user preferences are overlooked, resulting in model aging issue where model’s performance degrades along with time. To leverage dynamic characteristics, context enriched models are proposed to boost the performance and explainability of the recommender systems via modeling user’s temporal sequential behavior. However, most of them either only focus on modeling user’s sequential interactions within a path [6] or independently and separately of the recommendation mechanism [16]. More recently, Chen et al. [17] and Zhao et al. [18] explicitly leverage temporal item–item metapath and time-aware path reasoning; however, the key focus of these methods are mining temporal sequential feature for higher performance and interpretable recommendations. Some incremental learning and prequential evaluation frameworks are proposed to monitor evaluation metrics of general recommender systems as they continuously learn from a data stream [19]. However, incremental learning for explainable KG-based recommender systems needs further evaluation.

In this paper, we treat recommendation as an time-aware online prediction problem where data are split by time and old data are used for training while new data are used for inference; thus, the data leakage issue is eliminated. Moreover, we propose two model updating strategies to deal with the model aging issue, thus achieving high performance in long-term usage. In summary, we make the following three contributions:

- We point out two issues, namely the data leakage issue and the model aging issue, within existed explainable KG-based recommendations.
- We propose an updating enabled online prediction framework for time-aware explainable recommendation, including an online prediction scheme to eliminate the data leakage issue and two novel updating strategies to relieve the model aging issue.
- Extensive experiments are conducted on four real-world datasets. We simulate situations for both initial and long-term usage and validate recommendation accuracy and explainability. The experimental results demonstrate the lifelong superiority of our proposed methods.

The remainder of this paper is organized as follows: The related work is presented in Section 2. In Section 3, we introduce the motivations of our work. In Section 4, we describe the proposed approach. In Section 5, we discuss our experimental results. Finally, in Section 6, we conclude this paper.

## 2. Related Work

### 2.1. Explainable KG-Based Recommendation

Recent years have seen a surge in approaches that achieve the explainability of recommendations [1,20,21]. There are several different lines of research to build explainable recommendations; in this paper, we focus on explainable KG-based recommendations that are capable of leveraging knowledge graph embeddings as rich content information to enhance both of the recommendation performance and explainability [12,17]. Knowledge Graphs (KGs) as auxiliary data source, which contain background knowledge of items and their relations among them, have recently made significant contributions on recommender systems [22]. KGs are directed heterogeneous graphs in which nodes represent entities or attributes and edges represent relations. Numerous structured data are stored in KGs, where (h,r,t) denotes head entity *h* and tail entity *t* are linked by relation *r*. Thanks to rich structured information provided by knowledge graphs, the data sparsity issue and cold-start issue are alleviated; thus, superior recommendation performance is achieved. According to the methods used, KG-based recommendation are classified into three types, namely graph-based methods, path-based methods and embedding-based ones [23]. Path-based methods make use of metapaths first proposed by Gao et al. [5] to reason over KGs. However, it is impractical to enumerate all qualified metapaths in large-scale KGs [6,12]. Embedding-based methods focus on embedding knowledge graphs as latent vectors by using embedding models, such as TransE [24], DeepWalk [25], and node2vec [26], and then conducting similarity matching for recommendation. However, pure embedding-based methods are one-hop KG modeling approaches [27] and thus are not able to make recommendations containing multihop relational paths in KGs.

Besides enhancing recommendation performance, rich structured information within KGs are also used for recommendation explainability. Recently, Xian et al. [12] proposed a policy-guided path reasoning method to conduct explicit reasoning over KGs to make recommendations supported by an interpretable causal inference procedure. However, all the methods mentioned above dealt with recommendation as an offline prediction in which static KGs are used and models are trained once while used forever. To leverage dynamic characteristics, context enriched models are proposed to boost the performance and explainability of the recommender systems via modeling user’s temporal sequential behavior. However, most of them either only focus on modeling a user’s sequential interactions within a path [6] or independently and separately of the recommendation mechanism [16]. More recently, Chen et al. [17] explicitly model and leverage item–item metapath to improve the performance and explainability of the recommendation. Zhao et al. [18] proposed a time-aware reward to guide the reinforcement learning-based recommender. Unfortunately, these methods only mine temporal sequential feature for achieving higher performance while still ignore the model aging issue, i.e., achieving high performance in long-term usage.

### 2.2. Dataset Construction of Recommendation

Data construction of recommendation means a series of steps on preprocessing the original dataset and building the training and test sets. The impact of data construction strategies on recommendation performance was widely studied [13] In this section, we focus on data splitting and concept drift handling, which are the most relevant to our work.

**Data splitting.** The propose of data splitting is to divide the original dataset into training and test sets. As their significant impact of recommendation performance, different data splitting strategies of recommendation were widely evaluated [14,28]. And according to the choices of data ordering and splitting, there are mainly four types of them, including random ordering ratio-based splitting, random ordering leave-one-out splitting, temporal ordering ratio-based splitting, and temporal ordering leave-one-out splitting. Data ordering refers to arranging the interactions randomly or by a timestamp and splitting focus on the ratio of training and test sets. More recently, data leakage issue caused by not observing global timeline in recommender system has attracted increasing attention [15,29]. The damage of data leakage is disastrous, rendering the recommendations invalid, since future interactions are used to predict current user preference. To our best knowledge, however, the impact of data leakage on explainable recommendation is still ignored.

**Concept drift handling.** The interests of users or consumers change over time, and new topics may become popular, therefore resulting in interest shift [30] or concept drift [31]. Timely add the user’s new purchase information to the model, so as to master the user’s latest preferences, can improve the accuracy of recommendation [32,33]. These two facts emphasize the necessity and importance of model updating where old model retrains on new data. That is, a learning system is inevitable to be designed under the constraint of the stability–plasticity dilemma [34,35], which requires the learning system plasticity for the new knowledge while also requiring stability for the previous knowledge.

In this paper, we argue that the data leakage issue is due to random dataset split setting in offline prediction. Therefore, we transform the recommendation into an online prediction problem and present two novel model updating methods. According to the key idea of online prediction, that data are split by time and old data are used for training while new data are used for inference, data leakage issue is eliminated. Furthermore, we propose model updating strategies to enable the explainable recommender system dynamically adapt to new patterns of user preferences and operate without concern of model aging.

## 3. Motivations

To demonstrate the existing and impact of data leakage issue in explainable recommendation, we conduct two preliminary experiments. First, we make statistics on the temporal distribution of the training and test sets under cross validation on four Amazon real-world datasets, which are widely used in existing works [12,17], and observe serious data leakage phenomenon. As shown in Table 1, all the four datasets suffered from serious data leakage where knowledge should not be known at the time of training is utilized. Using CDs dataset for instance, training dataset and test dataset span all the ten years from 2005 to 2014. As a result, for instance, when making recommendation for user in 2005, the interaction information occurs after 2005 is used. In real-world recommendation, however, training and test dataset are strictly split by time and the test period is always after the training period. Therefore, future knowledge that is not expected to be available at the time of recommendation is utilized, thus data leakage occurs [29]. The impact of data leakage on recommendation results is widely studied, which causes high accuracy in cross validation but poor result on new data [13,15].

Second, we dive into the recommendation path of explainable recommendation results. The manifestation of information leakage on the path is that occurrence time of interaction in training set is later than that in test set. To this end, we conduct a three-step process as follows: (1) figure out interactions within inference path; (2) confirm the occurrence time of user–item interactions; (3) compare these occurrence time and check information leakage. According to the experimental setup in Section 5.3, the maximum path length is set to three, that is, there are four nodes in a path, where types of the first and last nodes are determined. The first node is the given user and the last node is one recommended item. Since there is no social information in the dataset, there is no connections between users. Moreover, users only interact with item and feature in KGs, so the second node in reference path must be item or feature. For the third node in reference path, there are two scenarios, i.e., user or others. As a result, given a reference path “n1-n2-n3-n4”, there exists two or four user–product interactions, depending on the type of node n3 is user. If the type of node n3 is attribute, there are two user–product interactions, including n1-n2, n1-n4. Otherwise, if the type of node n3 is user, there will be two more user–product interactions, namely n3-n2, n3-n4. Note that the interaction n1-n4 is recommendation result, and the other one or three interactions are extracted from training set. After figuring out interactions within inference path, we need to confirm the occurrence time of these interactions in training and test set, that is, to obtain one test occurrence time and one or three training occurrence times. Finally, we compare the test occurrence time and training occurrence times and information leakage is convicted when test occurrence time is not later than training occurrence times. The details of inaccurate explanations caused by data leakage are shown in Section 5.4.

## 4. The Proposed Methods

We propose an updating-enabled online prediction framework for time-aware explainable recommendation. The explainable recommendation component renders recommendation results as well as explanations, which we adopt widely used PGPR [12] as the base recommendation. To achieve the time awareness, we add two novel components, i.e., online prediction and model updating.

### 4.1. Online Prediction

**Figure 1:**
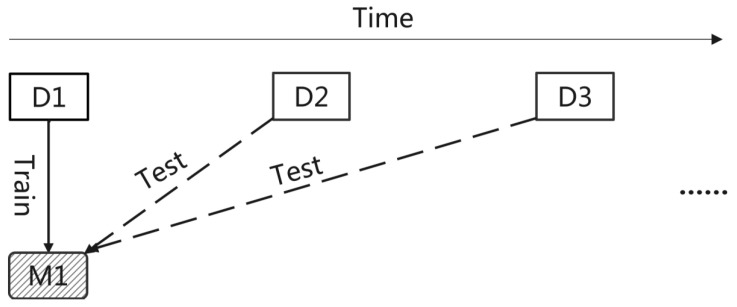
Online prediction, in which data are split by time and old data are used for training while new data are used for inference.

To deal with the data leakage issue, we formulate the recommendation problem as an online prediction problem instead of an offline prediction problem. That is, instead of randomly selecting data for training and test, which shuffles the natural sequence of the data, we simply do not shuffle data and pick a moment in time as the split point to divide training and test datasets. Specifically, all records collected before a given split point are used to train the model and all subsequent records are used as test data. Therefore, the training and test datasets will have no time overlap and the occurrence time of training dataset always precede that of test dataset. As shown in Figure 1, first of all, the total dataset is split by time into datasets D1, D2, and so on. Second, dataset D1 is used to train a recommender system M1, and as time goes by, the trained recommender system M1 is evaluated on the following datasets, i.e., D2 and its successors, respectively. As a result, no knowledge of future is used to train a recommender system under online prediction mode, thus eliminating the data leakage issue.

### 4.2. Model Updating Enabled Online Prediction

As mentioned above, a learning system must be designed to remain stable and unchanged to irrelevant events, while adaptive to new and important data, i.e., stability–plasticity dilemma [34,35]. However, whether in online prediction or offline prediction, a fixed training dataset is used to build the recommender system. These fixed methods are one extreme of the stability–plasticity dilemma that only considers stability, not plasticity. The latest interests of users, usually hidden in the newest data, however, are always ignored, resulting in model aging.

**Figure 2:**
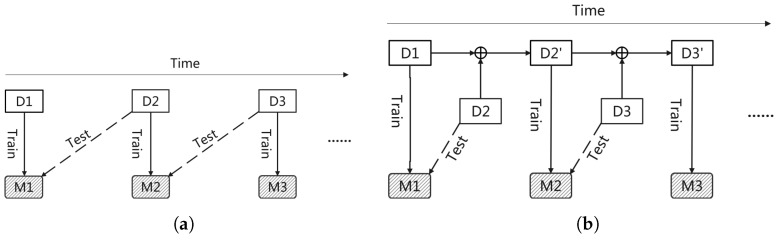
Model updating enabled online prediction, including (**a**) replacing-updating and (**b**) accumulation-updating. Models are retrained on modified training set.

To balance stability and plasticity within recommender systems, we propose to update models on modified training sets. Specifically, we propose two model updating strategies, the replacing-updating strategy and the accumulation-updating strategy, as shown in Figure 2. At the very beginning, same with online prediction, an initial training dataset D1 is collected and a recommender system M1 is trained on it. As with goes by, in replacing-updating, training dataset is replaced by the new coming data within one updating cycle and the old recommender system M1 is replaced by the new recommend system M2 trained on that modified training dataset D2, and so on. In accumulation-updating, new coming data with the latest updating cycle is add to the original training dataset D1 and new RS M2 is trained on accumulated training dataset $D2^{{}^{\prime }}$, and so on. Therefore, the main difference between these two model updating strategies is their ways of modifying training set.

The detailed replacing-updating algorithm and accumulation-updating algorithm are shown in Algorithms 1 and 2, respectively. As we can see, they both have three phases, including the initial training phase, updating phase, and inference phase. It is worth noting that, besides the difference of modified training set in updating phase mentioned above, another difference between these two model updating methods is in initial training phase. In initial training phase, training set in replacing-updating is data collected within last cycle before training, while that in accumulation-updating is all of data collected from the beginning. Thus, the training dataset in replacing-updating method is less than that in fixed method and accumulation-updating method.
**Algorithm 1:** Replacing-updating Enabled Online Recommendation**Require:** 
Initial training set $D_{0}$, updating cycle *T*, new received data during updating cycle $D_{T}$**Ensure:** 
Recommender system RS, Recommendations with explanations: Results1://Initial training phase2:$D\leftarrow D_{0}$3:RS←train(D)4://Updating phase5:**for** each cycle *T* **do**6:    $D\leftarrow D_{T}$7:    RS←train(D)8:**end for**9://Inference phase10:**for** each user *u* **do**11:    Results←recommend(RS,u)12:**end for**

In summary, fixed method and replacing-updating method are two extremes of the stability–plasticity dilemma. The fixed strategy gists the long-term preferences while ignores the instantaneous intents. On the contrary, the replacing-updating strategy focuses on the instantaneous intents while overlooking the long-term preferences. Finally, the accumulation-updating strategy is proposed as a compromise between the fixed strategy and the replacing-updating method, which gives considerations to both the long-term preferences and the instantaneous intents.
**Algorithm 2:** Accumulation-updating Enabled Online Recommendation**Require:** 
Initial training set $D_{0}$, updating cycle *T*, new received data during updating cycle $D_{T}$**Ensure:** 
Recommender system RS, Recommendations with explanations: Results1://Initial training phase2:$D\leftarrow D_{0}$3:RS←train(D)4://Updating phase5:**for** each cycle *T* **do**6:    $D\leftarrow D\cup D_{T}$7:    RS←train(D)8:**end for**9://Inference phase10:**for** each user *u* **do**11:    Results←recommend(RS,u)12:**end for**

## 5. Experiments

In this section, we extensively evaluate the effectiveness of our proposed method on four real-world datasets. We first introduce the datasets and preprocess, metrics as well as setup for experiments. Then, we evaluate the effectiveness of online prediction and model updating components. The aim is to answer the following two research questions (RQs):

- **RQ1.** How effective is the proposed online prediction method?
- **RQ2.** How effective is the proposed model updating method?

Besides the effectiveness, another concern of the proposed methods is the maintenance costs of the newly added online prediction and model updating components. For the online prediction component, it just changes the way of data splitting which is also included in the baseline model; thus, no extra maintenance costs are incurred. For the model updating component, compared to the fixed strategy, our proposed updating strategies involve two extra processes, including gathering new data for modifying training sets and retraining models on these modified training sets. These two extra processes bring about additional maintenance costs, namely storage costs and computational costs. For the replace-updating strategy, the additional maintenance costs in each updating cycle are constant, since the size of modified training set is stationary. As a contrast, the accumulation-updating strategy suffers from relatively large additional maintenance costs which scales linearly with the time length. That is, the additional maintenance costs of the proposed updating strategies are proportional to the updating interval. Note that in our implementation on four real-world datasets, the model updating interval is set as one year, which is relatively infrequent, to ensure sufficient new collected data. The data gathering and model updating processes are conducted offline; thus, they will not affect the online recommendation. As a result, compared to the fixed strategy, the additional maintenance costs of the proposed updating strategies are trivial.

### 5.1. Datasets Description and Preprocess

**Figure 3:**
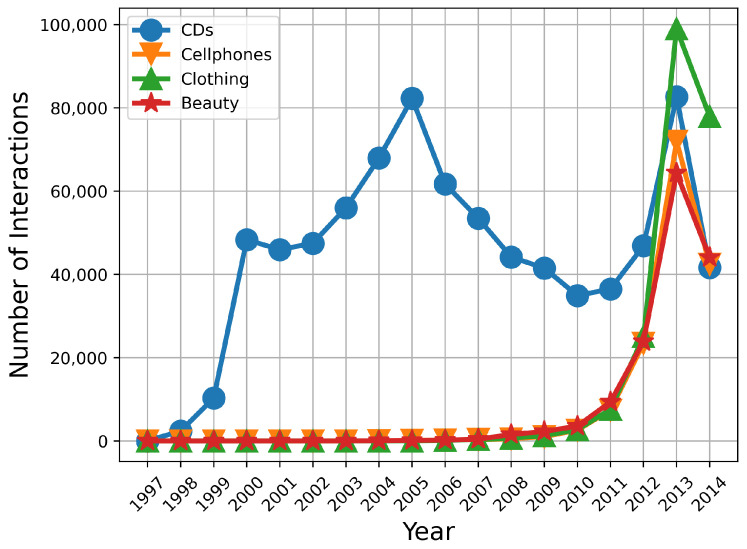
Distributions of datasets in years.

To evaluate the proposed method, we use publicly available real-world datasets from the Amazon e-commerce datasets collection [36], which contains products reviews and meta information. From the datasets, we select four categories, including CDs and Vinyl, Clothing, Cell Phones, and Beauty, which are used most commonly. The distributions of these four datasets in years are shown in Figure 3. As we can see, except for dataset CDs, the other datasets’ sizes are relatively small in the early stage. The description and statistics of four datasets are shown in Table 2. As we can see, apart from user and item entities, the item attributions are also considered for KGs building, including feature, brand, and category. To ensure sufficient training data, we take 2010 as the dividing line and split the remaining data by year. That is, data collected before 2010 are used as the initial training set for the fixed method and accumulation-updating method. Moreover, data collected after 2010 are used to update the modified training set in an annual cycle.

### 5.2. Metrics

We use four representative top-N recommendation measures to evaluate the effectiveness of recommendation, including Normalized Discounted Cumulative Gain (NDCG), Recall, Hit Ratio (HR), and Precision (Prec.).

NDCG is normalized DCG and calculated as following:

(1) $$\begin{array}{c} NDCG=\frac{\sum_{u\in U}\left|DCG\left(u\right)/IDCG\left(u\right)\right|}{\left|U\right|} \end{array}$$

where DCG(u) is a weighted sum of relevancy degree of ranked recommendations, IDCG(u) is DCG(u) measure of the ideal ranking results, and |U| is the number of users in test dataset. The details of NDCG refers to [37].

Recall is defined as the ratio of relevant recommendations to all the possible relevant items:

(2) $$\begin{array}{c} Recall=\frac{\sum_{u\in U}\left|\left(R\left(u\right)\cap T\left(u\right)\right)/T\left(u\right)\right|}{\left|U\right|} \end{array}$$

where R(u) is the recommendations, T(u) is the interested items to user *u*, and R(u)∩T(u) is the number of relevant items found in the recommendations.

Hit Ratio is defined as the proportion of users who are correctly recommended:

(3) $$\begin{array}{c} HR=\frac{\sum_{u\in U}Cap(|R\left(u\right)\cap T\left(u\right)|)}{\left|U\right|} \end{array}$$

where the function Cap(|R(u)∩T(u)|) is calculated as following:

(4) $$\begin{array}{c} Cap(|R\left(u\right)\cap T\left(u\right)|)=\left\{\begin{array}{cc} 0\;; & if|R(u)\cap T(u)|==0\\ 1\;; & if|R(u)\cap T(u)|\;!=0 \end{array}\right. \end{array}$$

Precision is defined as the ratio of relevant recommendations to the total provided recommendations:

(5) $$\begin{array}{c} Precision=\frac{\sum_{u\in U}|\left(R\left(u\right)\cap T\left(u\right)\right)/R\left(u\right)}{\left|U\right|} \end{array}$$

Note that these ranking metrics are computed based on the top-10 predictions for every user in the test dataset, which is widely used [6,12]. We calculated these four metrics in each model updating interval.

### 5.3. Experimental Setup

For all three methods, to train and evaluate the recommendation models practically and fair, as described in our online prediction, we divide the dataset into training and test sets according to time rather than randomly. The difference comes from the setup of model updating. For the replacing updating method (i.e., modifying training dataset with fixed-size sliding window [31]), we use the data collected within each cycle to build a new recommendation model. For the accumulation-updating method, we use the data collection before current cycle to build a new recommendation model. In both updating methods, we discard the old model and build new model to catch user least preferences. As a contrast, old model is always used without updating in baseline, i.e., no-updating method.

It is worth noting that, the base recommender system is adopted from an existing recommender system [12] for the evaluation. We adopt the same experimental parameters as in that work, which sets the maximum path length to 3 based on the assumption that shorter paths are more convincing. The readers are kindly referred to the original work [12] for more information about parameter settings.

### 5.4. The Effectiveness of Online Prediction

In this section, we extensively evaluate our proposed online prediction approach, providing a series of qualitative as well as quantitative analyses on four real-world datasets. The superiority of online prediction is mainly reflected in more practical results and more convincing explanations. In hand-out setting, the training dataset and test dataset are randomly selected from the total dataset, resulting in data leakage where future information is used to predict historical data. However, in the real world, the interaction data arrive in temporal order. Therefore, the recommender results and explanations of our proposed online prediction method will be more convincing. The impact of data leakage is recognized by existing works [14,15], but we are the first to offer a comprehensive critical study on this issue under the explainable recommendation scenario.

**Qualitative Analyses.** To intuitively understand how our model interprets the recommendation, we give a case study here based on the results generated in the previous experiments. As mentioned above in Section 3, we first study the path patterns discovered by our model during the reasoning process, followed by various cases for recommendation. We compare recommendation path under the data leakage scenario and online prediction model.

**Figure 4:**
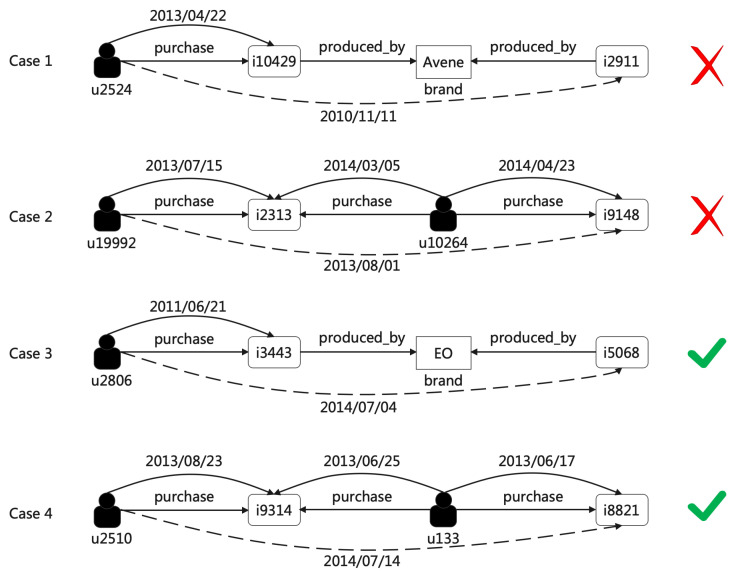
Cases of recommendation reasoning paths under offline prediction (case 1, case 2) and our online prediction (case 3, case 4).

As shown in Figure 4, we provide several real-world examples of the reasoning paths generated by offline prediction and online prediction. The first example (Case 1) comes from the Beauty dataset, where a user u2524 purchased an item i10429 which was produced by brand “Avene”. Meanwhile, another item i2911 was also produced by “Avene”. Therefore, i2911 was recommended to this user. In the second example (Case 2), there are two users, u19992 and u19264, who both purchased the item i2313, and user u10264 also purchased item i9148, which is one kind of collaborative filtering. So, item i9148 was recommended to the user u19992. These two recommendation cases are correct if the time factor is ignored. When considering the training time and inference time, however, these two recommendations are unrealistic and unreasonable in the real world. In case 1, the inference time is 11 November 2010 while the training time is 22 April 2013. In case 2, there are three “user–item” connections, whose time are 15 July 2013, 5 March 2014, and 23 April 2014, while the inference time is 1 August 2013. In other words, data leakage occurs, future data are used to build model to recommender for the past.

As a contrast, the recommendation paths in Case 3 and Case 4 are reasonable. In the third example (Case 3), a user u2806 bought an item i3443, which was produced by brand “EO”, which also produced item i5068. The last example (Case 4) also depicts user-based collaborative filtering; user u2510 and user u133 were regarded as neighbors, as they both purchased item i9314. Therefore, user u2510 was recommended item i8821, which was also purchased by user u133.

**Quantitative Analyses.** To examine to what extent the recommendations are invalid, we conduct quantitative analyses on the degree of recommendations validity under data leakage scenarios. As shown in Table 3, most of the recommendations are invalid; that is, there exists contradiction in time within the explainable recommendation paths. Statistics indicate the prevalence of data leakage in training and test sets. Supposing the training and test sets have size of *m* and *n*, respectively, and there are $k_{i}$ interactions in training set occur later than test interaction *i*. Then, the prevalence of data leakage is computed as $\frac{\sum_{i=1}^{n}k_{i}}{m\times n}$. It is obvious that the recommendation validity is approximately proportional to the prevalence of data leakage. This is easy to explain, since contradictions in time within recommendation paths have more chance to happen under serious data leakage. To this end, in our proposed online prediction, datasets are split along with time; thus, no future data are used to build the model. Therefore, we conclude that our online prediction method can eliminate data leakage as well as achieve reasonable recommendation results.

### 5.5. The Effectiveness of Model Updating

To estimate the effectiveness of proposed model updating strategies, we compare models trained with updating and without updating process. Model trained without updating refers to fixed model that is trained on data collected since the very beginning year and remains unchanged all the time. In contrast, models trained with updating, including replacing-updating and accumulation-updating, indicate models that need to retrain on the modified training dataset.

We compare our proposed methods with baseline on four Amazon real-world datasets. The results are reported in percentage and are calculated based on the top-10 predictions in the test set. The overall results are reported in Table 4. Note that the best results are highlighted in bold and the second-best results are underlined. As we can see, our model updating method outperforms the baseline on all of the four datasets of NDCG, Hit Rate, Recall and Precision. Specifically, accumulation-updating models achieve best results on three out of four datasets, and replacing-updating achieves the best results on the last CDs dataset. This shows the effectiveness of our proposed model updating strategies. It is worth noting that we just set the updating cycle, i.e., one year, intuitively; it might be possible to get better results with another carefully selected updating cycle.

**Accuracy over time.** Besides coverall recommendation results, we also conduct fine-grained evaluation of the proposed updating strategies. Specifically, we monitor the evolution of recommendation accuracy over time. One valuable feature of this fine-grained evaluation is that it allows examination of their effectiveness in the face of model aging. Under model aging, the performance of recommender degrades along with time. If the recommendation accuracy always maintains at a high level, then we can say model aging is relieved.

**Figure 5:**
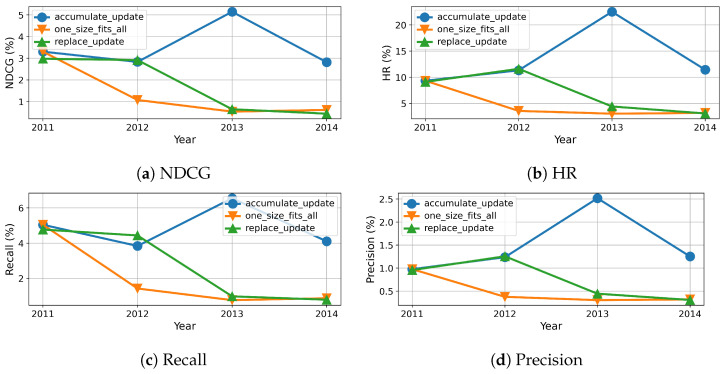
Recommendation effectiveness of our method compared to baseline on Beauty dataset.

**Figure 6:**
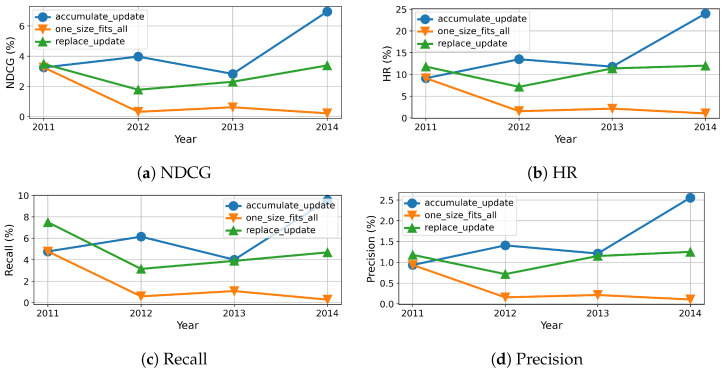
Recommendation effectiveness of our method compared to baseline on Cell dataset.

**Figure 7:**
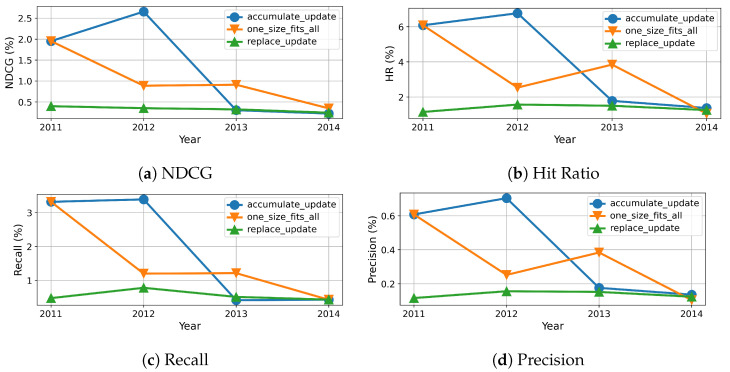
Recommendation effectiveness of our method compared to baseline on Cloth dataset.

**Figure 8:**
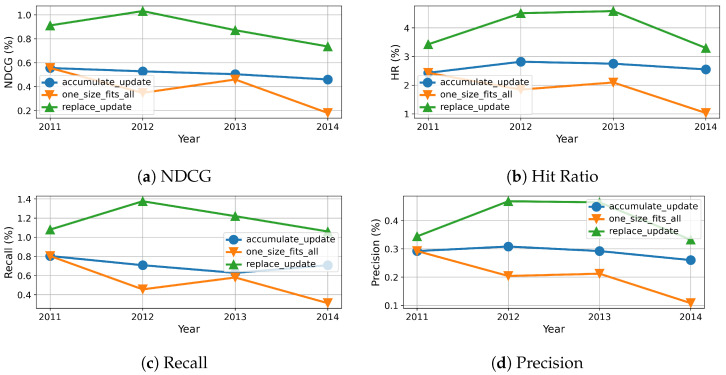
Recommendation effectiveness of our method compared to baseline on CD dataset.

To this end, we measure the evolution of results over time, which are reported in percentage and shown in Figure 5, Figure 6, Figure 7 and Figure 8. As we can see, the evolution of these three methods with each dataset generally confirms the overall results shown in Table 4, however more details become available. For example, the results of beginning of accumulation-updating method and fixed method are the same. The reason it that they are trained on the same dataset collected from the first year. For replace-updating, however, just data collected within the nearest year are used to train model.

Besides that, we also draw several interesting observations. First, the performance fluctuates over time for all the three methods with all datasets on all metrics. This phenomenon is because of the inherent volatility within the data, i.e., there exist significant differences between the number of users and items in each cycle. Second, the baseline methods, i.e., without model updating, suffer serious model aging that the recommender performance degrades along with time. This is because baseline methods just train once on dataset collected not after year 2011, the recommender can not adapt to the newest user preferences. Third, the performance of replace updating is sensitive to the dataset. For the Clothing dataset, as shown in Figure 7, replace updating is the worst. One possible reason is that clothing fashion has some cycle, and replace updating just preserves the newest user preferences and ignores the old fashion.

Fourth, when comparing the replace updating with accumulate updating, the accumulate updating outperforms replace updating in three datasets. One possible reason is that accumulate updating use more data than replace updating. As a result, accumulate updating can preserve long-term preference while absorbing new interest of users. One exception is the CDs dataset, as shown in Figure 8, the results of replace updating are better then those of accumulate updating. One possible reason is that CDs are gradually replaced by streaming media; the past preferences have no enlightenment on current preferences. Fifth, when comparing model updating methods with baseline no-updating method, our model updating methods always show superior results than the baseline method. Moreover, the stability of models with updating are also superior to the ones without updating. One possible reason is that the no-updating models are trained with only the samples collected not after year 2011, which hinders their adaption to the continuous update of forthcoming data. As a result, these results suggest that model updating is necessary and effective in recommendation systems.

## 6. Conclusions

In this paper, we propose a novel model updating-enabled online prediction method for knowledge graph-based recommendation that can effectively address the issues of data leakage and model aging. Our time-aware proposed method treats recommendation as a online prediction problem; thus, the data leakage issue rooted in random dataset split setting within offline learning is eliminated. Moreover, two model updating strategies are introduced to deal with the model aging issue. Experimental results on four real-world datasets demonstrate, compared with the state-of-the-art, our approach achieve higher accuracy as well as more convincing explanations for the entire lifetime of recommendation systems, i.e., both the initial period and the long-term usage. It should be noted that our updating enabled online prediction approach is a flexible recommendation framework and can be extended to many other recommender algorithms, which will be explored in the future.

## Figures and Tables

**Table 1:** Temporal data distribution of four Amazon real-world datasets under hand-out cross validation.

Datasets		Year									
		≤2005	2006	2007	2008	2009	2010	2011	2012	2013	2014
CDs	training	360,695	61,696	53,484	44,168	41,509	34,917	36,482	46,862	82,653	41,624
	test	133,814	23,078	19,713	16,258	15,294	12,780	13,213	16,895	28,389	14,067
	total	494,509	84,774	73,197	60,426	56,803	47,697	49,695	63,757	111,042	55,691
Cellphones	training	130	196	312	525	1011	2,602	7385	23,452	72,002	42,433
	test	48	44	77	152	296	829	2206	6890	21,237	12,612
	total	178	240	389	677	1307	3431	9591	30,342	93,239	55,045
Clothing	training	30	98	342	661	1266	2688	7575	25,085	99,007	77,944
	test	5	17	109	207	395	770	2271	7569	29,511	23,127
	total	35	115	451	868	1661	3458	9846	32,654	128,518	101,071
Beauty	training	173	169	402	1540	2284	3551	9352	23,892	64,382	44,099
	test	54	53	137	545	768	1206	2965	7851	20,869	14,210
	total	227	222	539	2085	3052	4757	12,317	31,743	85,251	58,309

**Table 2:** Dataset Description and Statistics.

Datasets	Entities					Year				
	User	Item	Feature	Brand	Category	≤2010	2011	2012	2013	2014
CDs	75,258	64,443	202,959	1414	770	817,406	49,695	63,757	111,042	55,691
Clothing	39,387	23,033	21,366	1182	1193	6222	9591	30,342	93,239	55,045
Cellphones	27,879	10,429	22,493	955	206	6588	9846	32,654	128,518	101,071
Beauty	22,363	12,101	22,564	2077	248	10,882	12,317	31,743	85,251	58,309

**Table 3:** Validaty of Recommendations and Prevalence of Data Leakage Statistics.

Metrics	Offline Prediction				Online Prediction
	CDs	Clothing	Cellphones	Beauty	
validity of recommendations	52.02%	43.76%	38.81%	40.29%	100%
prevalence of data leakage	3.00%	5.14%	5.67%	5.02%	0%

**Table 4:** Overall recommendation effectiveness of our method compared to baseline on four Amazon real-world datasets. The results are reported in percentage (%) and are calculated based on the top-10 predictions in the test set. The best results are highlighted in bold and the second-best results are underlined.

Datasets	Methods	Measures(%)			
		NDCG	Recall	HR	Prec.
Beauty	fixed (baseline)	1.384	2.028	4.788	0.492
	replacing-updating	1.744	2.744	7.060	0.740
	accumulation-updating	**3.524**	**4.884**	**13.660**	**1.496**
Cellphones	fixed (baseline)	1.104	1.680	3.460	0.352
	replacing-updating	2.740	4.808	10.584	1.076
	accumulation-updating	**4.252**	**6.120**	**14.600**	**1.528**
Clothing	fixed (baseline)	1.024	1.544	3.380	0.336
	replacing-updating	0.332	0.556	1.368	0.136
	accumulation-updating	**1.284**	**1.892**	**4.000**	**0.404**
CDs	fixed (baseline)	0.388	0.536	1.852	0.204
	replacing-updating	**0.888**	**1.184**	**3.956**	**0.404**
	accumulation-updating	0.512	0.712	2.640	0.288

## Data Availability

Not applicable.
